# Comprehensive identification, characterization, and expression analysis of the *MORF* gene family in *Brassica napus*

**DOI:** 10.1186/s12870-024-05177-3

**Published:** 2024-05-30

**Authors:** Jiani Xing, Yayi Zhang, Wenjian Song, Nadia Ahmed Ali, Kexing Su, Xingxing Sun, Yujia Sun, Yizhou Jiang, Xiaobo Zhao

**Affiliations:** Key Laboratory of Nuclear Agricultural Sciences of Ministry of Agriculture and Rural Affairs, Key Laboratory of Nuclear Agricultural Sciences of Zhejiang Province, Institute of Nuclear Agricultural Sciences, College of Agriculture and Biotechnology, Zhejiang University, Hangzhou, 310058 China

**Keywords:** *MORF* gene family, RNA editing, Phylogenetic analysis, Gene expression, *Brassica napus*

## Abstract

**Background:**

RNA editing in chloroplast and mitochondrion transcripts of plants is an important type of post-transcriptional RNA modification in which members of the multiple organellar RNA editing factor gene family (MORF) play a crucial role. However, a systematic identification and characterization of *MORF* members in *Brassica napus* is still lacking.

**Results:**

In this study, a total of 43 *MORF* genes were identified from the genome of the *Brassica napus* cultivar “Zhongshuang 11”. The *Brassica napus* MORF (BnMORF) family members were divided into three groups through phylogenetic analysis. *BnMORF* genes distributed on 14 chromosomes and expanded due to segmental duplication and whole genome duplication repetitions. The majority of BnMORF proteins were predicted to be localized to mitochondria and chloroplasts. The promoter *cis*-regulatory element analysis, spatial-temporal expression profiling, and co-expression network of *BnMORF* genes indicated the involvement of *BnMORF* genes in stress and phytohormone responses, as well as growth and development.

**Conclusion:**

This study provides a comprehensive analysis of *BnMORF* genes and lays a foundation for further exploring their physiological functions in *Brassica napus*.

**Supplementary Information:**

The online version contains supplementary material available at 10.1186/s12870-024-05177-3.

## Background

As a post-transcriptional RNA metabolic process, RNA editing generally modifies the genetic information present in RNA molecules through nucleotide insertion, deletion, or conversion [1]. In plants, RNA editing occurs mainly in the two organelles with resident genomes: chloroplast and mitochondrion, involving the cytidine to uridine (C-to-U) modification (rarely U-to-C) within coding or noncoding regions of RNAs [2, 3]. There are about 20 to 40 and 400 to 600 conserved C-to-U editing sites in the chloroplast and mitochondrion genome of most flowering plants, respectively [4]. In plants, RNA editing plays important roles in organelle biogenesis, organelle signaling, and adaptation to environmental stresses. Previous studies showed that many mutant plants with impaired site-specific RNA editing exhibited strong deleterious phenotypes, even lethal ones [2, 5–8].

In plants, the RNA editing process is conducted by molecular machinery called the RNA editosome, which is formed by various nuclear-encoded RNA editing factors. These factors include pentatricopeptide repeat proteins (PPR), multiple organellar RNA editing factor (MORF) proteins/RNA editing factor interacting proteins (RIPs) [3, 9, 10], chloroplast ribonucleoproteins (cpRNPs), organelle RNA recognition motif proteins (ORRM) [11, 12], organelle zinc-finger proteins (OZ) [13], and protoporphyrinogen oxidase 1 (PPO1) [14]. In RNA editosomes, PPR proteins bind to RNA molecules and recognize RNA editing sites. The PPR-RNA complex is structured into editosomes together with many non-PPR protein factors. These non-PPR proteins, including MORF and ORRM proteins, might play as connectors of site-specific PPR proteins with other RNA editing factors or editing efficiency regulators. There are ten MORF family members in *Arabidopsis thaliana*. MORF2 and MORF9 are chloroplast-localized, MORF8 is dual-localized in the mitochondrion and chloroplast, and the remaining MORF proteins are mitochondrion-localized. Five members of the MORF family act as key RNA editing factors in mitochondria and/or chloroplasts, whereas the rest of the MORFs have less or no impact on RNA editing [9, 10]. It was reported that disrupting the *MORF1*, *MORF3*, and *MORF8* genes decreased the editing efficiency at 19%, 26%, and 72% of mitochondrial RNA editing sites, respectively. The loss of function of MORF2 or MORF9 abolished the RNA editing efficiency at almost all chloroplast editing sites [10, 15].

Members of the MORF family have also been identified in several different plant species, including *Populus trichocarpa* with nine [5], *Oryza sativa* with seven [16], *Zea mays* with seven [17], and *Nicotiana benthamiana* with eight members [18]. MORF members contain a centrally conserved MORF domain composed of a core of six anti-parallel β-sheets flanked on one side by three α-helices and several loops on the other side [19, 20]. MORF proteins can form homodimers and heterodimers and selectively interact with other RNA editing factors, such as site-specific PPR proteins, ORRM proteins, and PPO1 [3]. Furthermore, MORF members may be essential to the growth, development, and stress responses of plants. In *Arabidopsis*, leaves of *morf2* and *morf9* mutants showed decreased chlorophyll content, and *morf8* mutant plants exhibited a dwarf phenotype [9, 21]. In rice, mutant plants of *WSP1* encoding a protein from the MORF family had reduced chlorophyll content and emerged with a white immature panicle phenotype [22], and the expression of rice *MORF* genes was affected by cold and salt treatments [16]. In tobacco, *NbMORF8* has been demonstrated to negatively regulate the immunity of plants to pathogens [18]. The poplar *PtrMORF* genes have also been found to respond to drought stress [5].

The key oil crop, *Brassica napus*, is grown extensively all over the world and is one of the most significant sources of sauces, vegetables, and industrial oil [23]. *Brassica napus* (AnAnCnCn, 2n = 38) developed naturally from the hybridization of two diploid species, *Brassica rapa* (*B. rapa*; AnAn, *n* = 10) and *Brassica oleracea (B. oleracea*; CnCn, *n* = 9) [24, 25]. However, the genome-wide identification and characterization of the *Brassica napus MORF* (*BnMORF*) gene family is still lacking. In this study, members of the *BnMORF* gene family were identified and analyzed, including their gene structures, chromosomal localizations, and evolutionary patterns. The expression profile of *BnMORF* genes in various tissues and under different phytohormone and stress treatments was explored. The *cis*-regulatory elements and transcription factor binding sites in promoters of *BnMORF* genes were also identified. This study will help us better understand the *MORF* gene family and establish a basis for future exploration and functional validation of *MORF* genes in *Brassica napus*.

## Results

### Identification and classification of the *Brassica napus MORF* (*BnMORF*) gene family members

To obtain all the candidate *Brassica napus MORF* gene family members, we performed a BLASTP homology search against the *Brassica napus* cultivar Zhongshuang 11 (ZS11) genome using *Arabidopsis* MORF protein sequences as queries. Subsequently, by confirming the presence of the MORF domain (IPR039206) in candidate BnMORF members and removing the redundant gene forms simultaneously, 43 *BnMORF* genes were genome-widely identified from the ZS11 genome (Table 1). All the identified *BnMORF* genes were then given new names according to their chromosome locations, and their gene IDs from different versions of genome annotation were also identified (Table 1). As shown in Table 2, the BnMORF proteins have a length ranging from 116 to 910 amino acids, and the average value was 297.44 amino acids. The isoelectric point (pI) of BnMORF proteins varies from 5.09 to 9.75, with an average value of 8.23. Their molecular weight (MW) varies from 13.56 to 98.84 kDa, with an average value of 32.91 kDa. Prediction of subcellular localization revealed that ten BnMORF proteins were identified in chloroplasts, while 27 BnMORF proteins are localized to mitochondria, and the remaining six proteins are localized elsewhere.

**Table 1:** The list of identified *BnMORF* genes in *Brassica napus* by this study

Gene name	Genome Location	+/-stand	Gene ID		
			ZS11.v10	ZS11_HZAU_V1.0	Darmor-bzh
*BnMORF1*	chrA01:6126416–6,128,630	-	ZS11A01G010980	BnaA01G0106900ZS	BnaA01g10360D
*BnMORF2*	chrA01:28849269–28,851,793	-	ZS11A01G038120	N/A	N/A
*BnMORF3*	chrA01:33834340–33,835,493	-	ZS11A01G045540	N/A	N/A
*BnMORF4*	chrA03:8606070–8,607,171	-	ZS11A03G017260	BnaA03G0169000ZS	BnaA03g16150D
*BnMORF5*	chrA03:16126716–16,128,834	-	ZS11A03G031220	BnaA03G0305400ZS	BnaA03g29680D
*BnMORF6*	chrA03:17982112–17,984,070	+	ZS11A03G034900	N/A	N/A
*BnMORF7*	chrA03:24743456–24,745,682	-	ZS11A03G046870	BnaA03G0455700ZS	BnaA03g44360D
*BnMORF8*	chrA04:18110516–18,112,517	+	ZS11A04G023540	BnaA04G0215000ZS	BnaA04g19490D
*BnMORF9*	chrA04:18638112–18,639,544	-	ZS11A04G024620	BnaA04G0225400ZS	N/A
*BnMORF10*	chrA05:215057–216,453	-	ZS11A05G000440	N/A	N/A
*BnMORF11*	chrA05:5469265–5,470,623	+	ZS11A05G009520	BnaA05G0093900ZS	BnaA05g08720D
*BnMORF12*	chrA05:6303789–6,306,719	-	ZS11A05G010990	BnaA05G0108000ZS	BnaA05g10140D
*BnMORF13*	chrA05:19277258–19,279,347	-	ZS11A05G024840	BnaA05G0347500ZS	BnaA05g19970D
*BnMORF14*	chrA05:23301144–23,315,760	-	ZS11A05G030820	BnaA05G0402900ZS	BnaA05g24520D
*BnMORF15*	chrA06:4300071–4,301,979	+	ZS11A06G007720	BnaA06G0073900ZS	BnaA06g07110D
*BnMORF16*	chrA06:28495250–28,498,223	-	ZS11A06G041400	BnaA06G0433100ZS	N/A
*BnMORF17*	chrA07:4188805–4,190,149	-	ZS11A07G005440	BnaA07G0043500ZS	N/A
*BnMORF18*	chrA07:22371357–22,372,620	-	ZS11A07G029000	BnaA07G0258700ZS	BnaA07g23020D
*BnMORF19*	chrA07:27027874–27,029,637	+	ZS11A07G036840	BnaA07G0333800ZS	BnaA07g30270D
*BnMORF20*	chrA08:21498045–21,500,070	-	ZS11A08G029760	BnaA08G0284200ZS	BnaA08g25290D
*BnMORF21*	chrC01:8959436–8,961,766	-	ZS11C01G013620	BnaC01G0130400ZS	BnaCnng18610D
*BnMORF22*	chrC01:47769564–47,771,109	-	ZS11C01G051320	BnaC01G0435100ZS	BnaC01g36450D
*BnMORF23*	chrC01:54055555–54,058,398	-	ZS11C01G060210	BnaC01G0489800ZS	N/A
*BnMORF24*	chrC03:10641039–10,642,556	+	ZS11C03G019700	BnaC03G0187800ZS	BnaC03g18650D
*BnMORF25*	chrC03:11144693–11,145,818	-	ZS11C03G020440	BnaC03G0195700ZS	BnaC03g19330D
*BnMORF26*	chrC03:24757588–24,759,584	-	ZS11C03G038800	BnaC03G0366800ZS	BnaC03g34900D
*BnMORF27*	chrC03:28069877–28,071,896	+	ZS11C03G043350	N/A	N/A
*BnMORF28*	chrC03:66602160–66,605,068	-	ZS11C03G079820	BnaC03G0717100ZS	BnaC03g63630D
*BnMORF29*	chrC04:10038313–10,039,776	+	ZS11C04G012530	BnaC04G0112500ZS	BnaC04g09860D
*BnMORF30*	chrC04:11892495–11,894,664	-	ZS11C04G015060	BnaC04G0135100ZS	BnaC04g11080D
*BnMORF31*	chrC04:61023156–61,025,197	+	ZS11C04G063850	BnaC04G0526200ZS	BnaC04g43880D
*BnMORF32*	chrC04:61782498–61,783,939	-	ZS11C04G065140	BnaC04G0538600ZS	N/A
*BnMORF33*	chrC05:4976241–4,977,722	+	ZS11C05G009080	BnaC05G0091500ZS	BnaCnng17940D
*BnMORF34*	chrC05:41571267–41,573,381	-	ZS11C05G044530	BnaC05G0377600ZS	BnaC05g31350D
*BnMORF35*	chrC05:49351755–49,354,071	-	ZS11C05G053380	N/A	N/A
*BnMORF36*	chrC06:37014945–37,016,303	-	ZS11C06G035830	BnaC06G0288800ZS	N/A
*BnMORF37*	chrC06:37056571–37,057,469	-	ZS11C06G035910	BnaC06G0289100ZS	BnaC06g24260D
*BnMORF38*	chrC06:46276010–46,277,392	+	ZS11C06G047270	BnaC06G0392000ZS	BnaC06g33720D
*BnMORF39*	chrC07:10634028–10,635,577	+	ZS11C07G008710	BnaC07G0066800ZS	N/A
*BnMORF40*	chrC07:12129412–12,130,634	-	ZS11C07G009940	BnaC07G0074600ZS	N/A
*BnMORF41*	chrC07:35565963–35,569,095	+	ZS11C07G029930	BnaC07G0234700ZS	BnaCnng36780D
*BnMORF42*	chrC07:51906862–51,909,612	-	ZS11C07G052450	BnaC07G0431700ZS	BnaC07g36190D
*BnMORF43*	chrC08:29677819–29,679,907	+	ZS11C08G025790	BnaC08G0208800ZS	BnaC08g14930D

**Table 2:** Protein features of the 43 BnMORF family members identified in *Brassica napus* cultivar ZS11

Gene name	Protein ID	Protein length (aa)	Subcellular localization	cTP	mTP	Molecular weight (Da)	pI
*BnMORF1*	GWHPANRE001886	383	mito		Y	41201.27	8.7
*BnMORF2*	GWHPANRE004883	379	chlo	Y		40906.46	9.5
*BnMORF3*	GWHPANRE005697	247	mito		Y	27908.37	9.08
*BnMORF4*	GWHPANRE012935	137	other			16,435	9.26
*BnMORF5*	GWHPANRE014575	244	mito		Y	27590.04	9.05
*BnMORF6*	GWHPANRE015004	389	mito		Y	42230.96	9.6
*BnMORF7*	GWHPANRE016352	374	mito		Y	40619.71	6.96
*BnMORF8*	GWHPANRE020847	222	chl	Y		24955.09	7.56
*BnMORF9*	GWHPANRE020960	229	mito		Y	26012.39	9.3
*BnMORF10*	GWHPANRE021862	215	mito		Y	23214.13	5.76
*BnMORF11*	GWHPANRE022875	234	mito		Y	26464.83	8.99
*BnMORF12*	GWHPANRE023040	230	chl	Y		25709.93	6.83
*BnMORF13*	GWHPANRE024570	373	chl	Y		41359.85	6.11
*BnMORF14*	GWHPANRE025252	910	mito		Y	98841.03	7.09
*BnMORF15*	GWHPANRE027369	230	chl	Y		25779.15	9.23
*BnMORF16*	GWHPANRE031195	699	mito		Y	76083.65	6.63
*BnMORF17*	GWHPANRE032039	223	chl	Y		25414.87	9.17
*BnMORF18*	GWHPANRE034719	163	mito		Y	18227.09	9.1
*BnMORF19*	GWHPANRE035605	190	mito		Y	21904.99	9.2
*BnMORF20*	GWHPANRE039727	232	chl	Y		26362.75	9.11
*BnMORF21*	GWHPANRE052474	380	mito		Y	41060.2	8.5
*BnMORF22*	GWHPANRE056658	342	other			36519.8	7.12
*BnMORF23*	GWHPANRE057662	247	mito		Y	27776.27	9.03
*BnMORF24*	GWHPANRE067578	116	other			13564.14	5.62
*BnMORF25*	GWHPANRE067659	233	mito		Y	26567.27	9.75
*BnMORF26*	GWHPANRE069761	215	mito		Y	23975.27	9.02
*BnMORF27*	GWHPANRE070266	399	mito		Y	43159.9	9.53
*BnMORF28*	GWHPANRE074336	251	other			27454.92	5.09
*BnMORF29*	GWHPANRE077011	230	mito		Y	26094.42	9.21
*BnMORF30*	GWHPANRE077303	231	chl	Y		25750.99	6.74
*BnMORF31*	GWHPANRE082686	228	chl	Y		25562.69	8.52
*BnMORF32*	GWHPANRE082833	230	mito		Y	26043.45	9.3
*BnMORF33*	GWHPANRE084812	271	chl	Y		30124.19	8.98
*BnMORF34*	GWHPANRE088764	398	other			44228.17	6.8
*BnMORF35*	GWHPANRE089750	402	mito		Y	43357.21	9.35
*BnMORF36*	GWHPANRE094844	190	mito		Y	21453.36	9.3
*BnMORF37*	GWHPANRE094852	156	mito		Y	17135.81	7.66
*BnMORF38*	GWHPANRE096124	183	mito		Y	20860.06	9.26
*BnMORF39*	GWHPANRE097888	239	mito		Y	26891.61	8.76
*BnMORF40*	GWHPANRE098014	168	other			19325.81	5.85
*BnMORF41*	GWHPANRE100217	740	mito		Y	80249.44	7.31
*BnMORF42*	GWHPANRE102782	379	mito		Y	41071.24	7.63
*BnMORF43*	GWHPANRE107019	259	mito		Y	29581.58	9.45

Abbreviations: aa, amino acids; chl, chloroplast, mito, mitochondrion; cTP: chloroplast transit peptide; mTP: mitochondrial transit peptide; Y: yes, pI, isoelectric point.

### Phylogenetic and sequence structural features of BnMORF family members

**Fig. 1:**
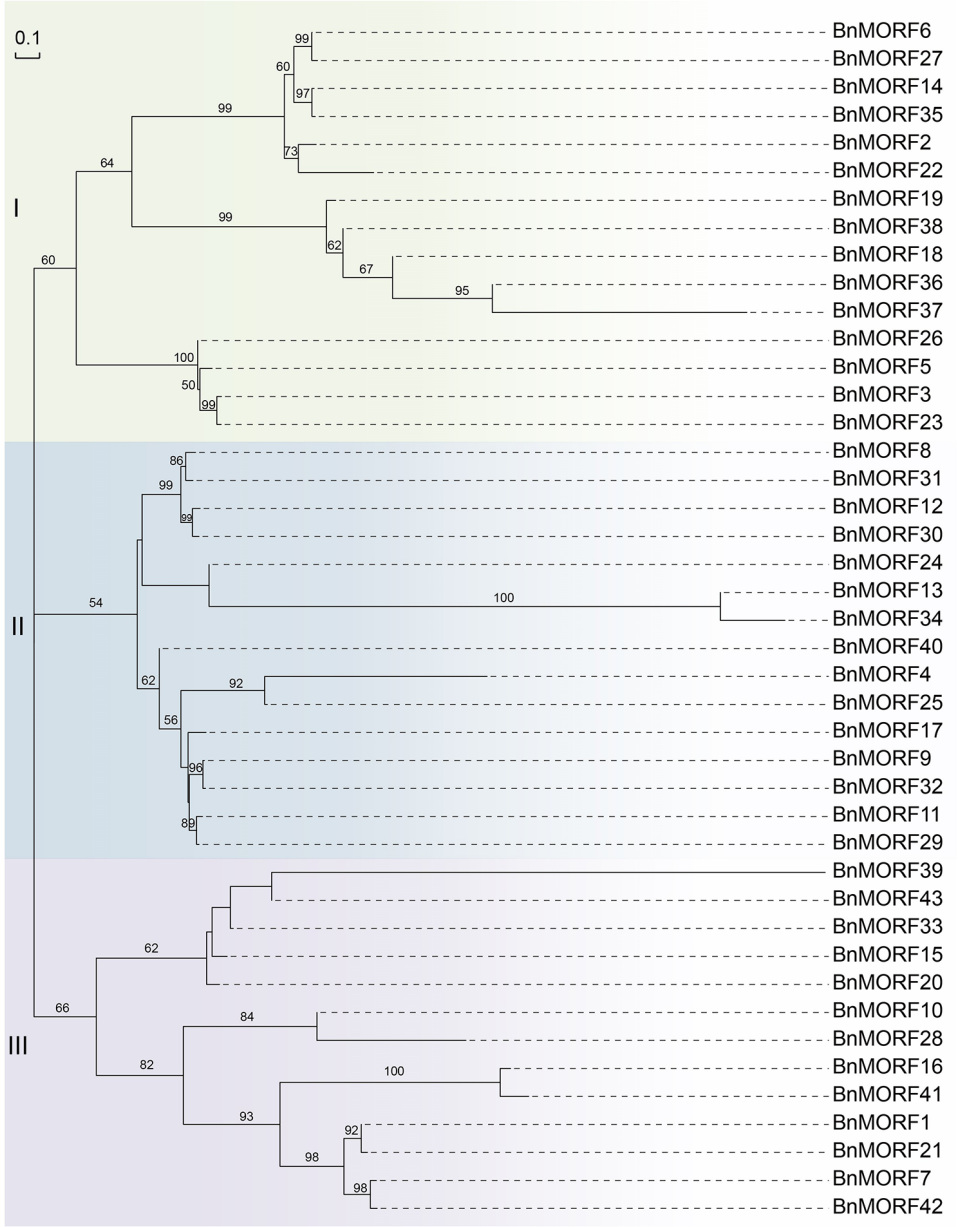
Neighbor-joining phylogenetic tree of BnMORF family members. The topology was assessed with a bootstrap analysis with 1000 replicates. All BnMORF members are divided into groups I–III. The bootstrap values are displayed, and various groups are denoted by distinct background colors. The length of the branches represents evolutionary distances, and the scale bar indicates 0.1 substitutions each point

To investigate the classification and evolutionary relationship of the MORF members, an unrooted phylogenetic tree using the neighbor-joining method was constructed from 67 MORF protein sequences collected from maize (*Zea mays*), *Arabidopsis thaliana*, *Brassica napus*, and rice (*Oryza sativa)* (Additional file 1: Fig. S1). Based on the phylogenetic tree, all the MORF proteins can be subdivided into three groups, named I to III. The size of the three groups varies. Group II is the largest group, containing 23 MORF members (Additional file 1: Fig. S1). After the lineage separation, species-specific gene duplications occurred, resulting in the inclusion of multiple *MORF* genes per species. The phylogenetic tree only containing the 43 BnMORF members showed that BnMORF members were also divided into three similar groups, according to homology relationships (Fig. 1). BnMORF members are equivalent in groups I and II, with 15 members, while group III has 13.

**Fig. 2:**
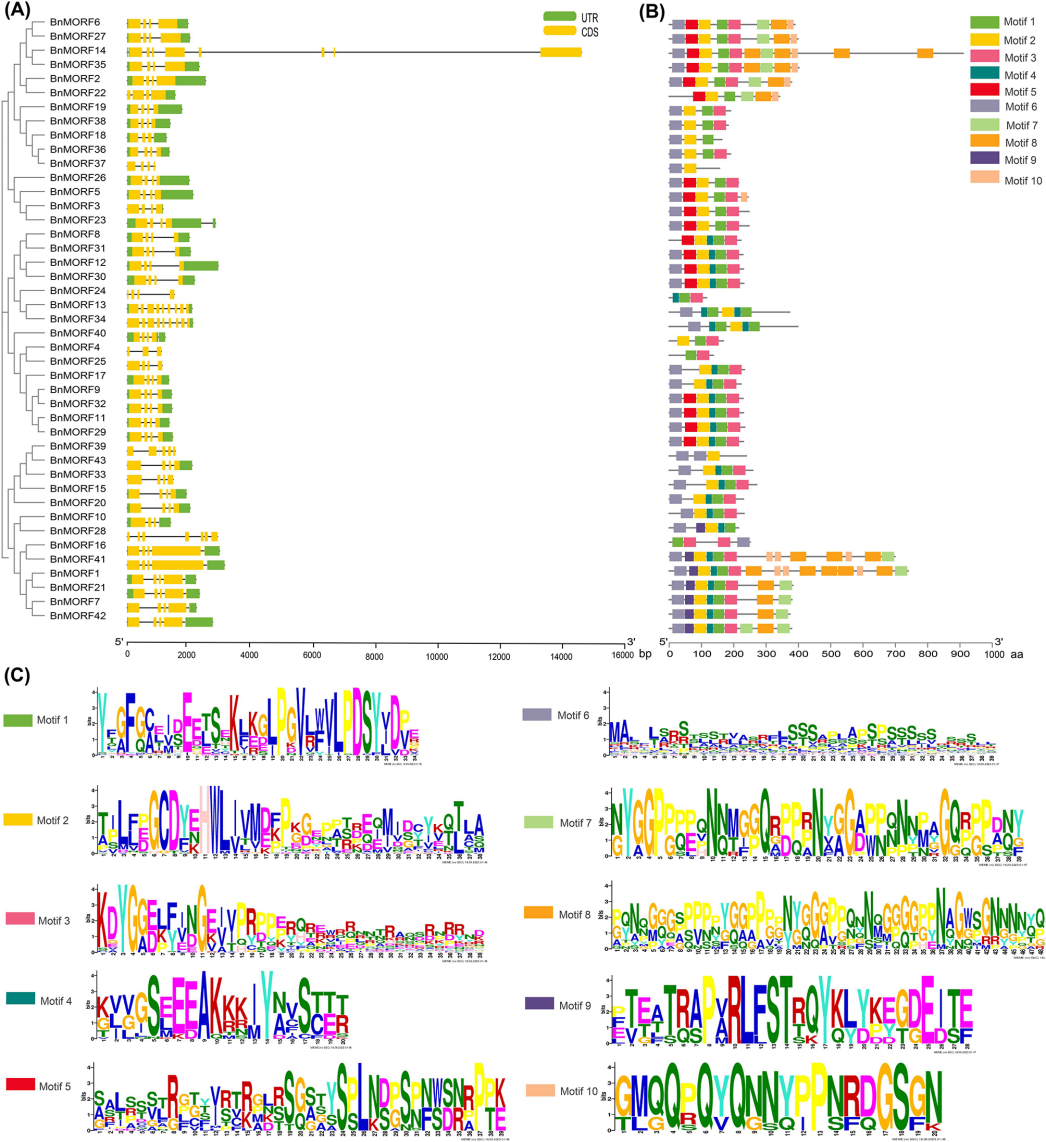
Comparison of conserved protein motifs and gene structures of BnMORF members. **(A)** Schematic exon/intron structures of *BnMORF* genes. The exons are indicated by yellow rectangles, and the introns are represented by black lines. The UTR regions are indicated by green rectangles. **(B)** Conserved motifs in BnMORF proteins. Ten motifs are indicated by different colored rectangles. **(C)** The sequence logos of the ten conserved motifs. The *x*-axis represents the conserved sequence of each domain. The *y*-axis represents a measure of relative entropy, and the height of each letter indicates the rate of conservation of each amino acid across all proteins

Exon-intron structural changes in genes and motif composition in proteins both play vital roles in gene/protein functional differentiation. Motif composition and the intron/exon structure of each BnMORF member were analyzed (Fig. 2). The *BnMORF* genes contained several exons ranging from three to nine. *BnMORF4* contained only three exons, while *BnMORF13* and *BnMORF34* contained nine exons (Fig. 2a). Most *BnMORF* genes within the same phylogenetic tree clan or in the same group presented similar exon-intron distribution patterns. We annotated conserved motifs in BnMORF proteins using the MEME server, and one to ten conserved motifs were found in BnMORF family members (Fig. 2b and c). All BnMORF proteins except BnMORF37 and BnMORF39 contain the highly conserved motif 1, which consists of 34 amino acids. Except for BnMORF4, BnMORF24, and BnMORF28, all the other BnMORF proteins have the 38-amino acid conserved motif 2 (Fig. 2b and c). Some motifs specifically exist in BnMORF members from the same phylogenetic tree clan, and they may link to unique biological functions.

### Chromosomal locations and collinearity analysis of *BnMORF* genes

**Fig. 3:**
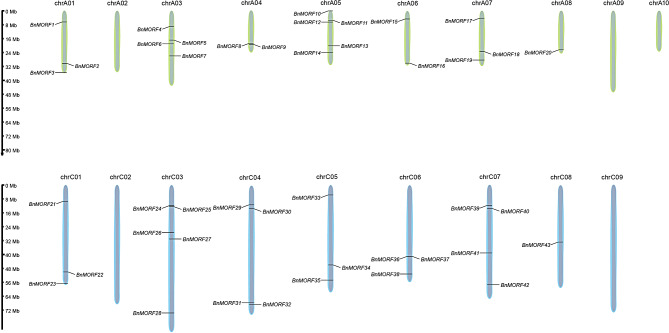
The chromosomal distribution of *BnMORF* genes. The chromosome numbers are indicated at the top of every chromosome, and each chromosome distance is displayed in megabytes (Mb) at the bottom. The *BnMORF* gene names are highlighted in black

**Fig. 4:**
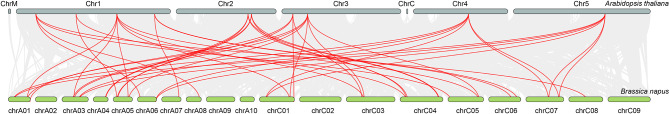
The synteny analysis of MORF family members between *Brassica napus* and *Arabidopsis*. The collinear blocks generated by the *Brassica napus* and *Arabidopsis thaliana* genomes are indicated with gray lines in the background, whereas syntenic *MORF* gene pairs are indicated with red lines

**Fig. 5:**
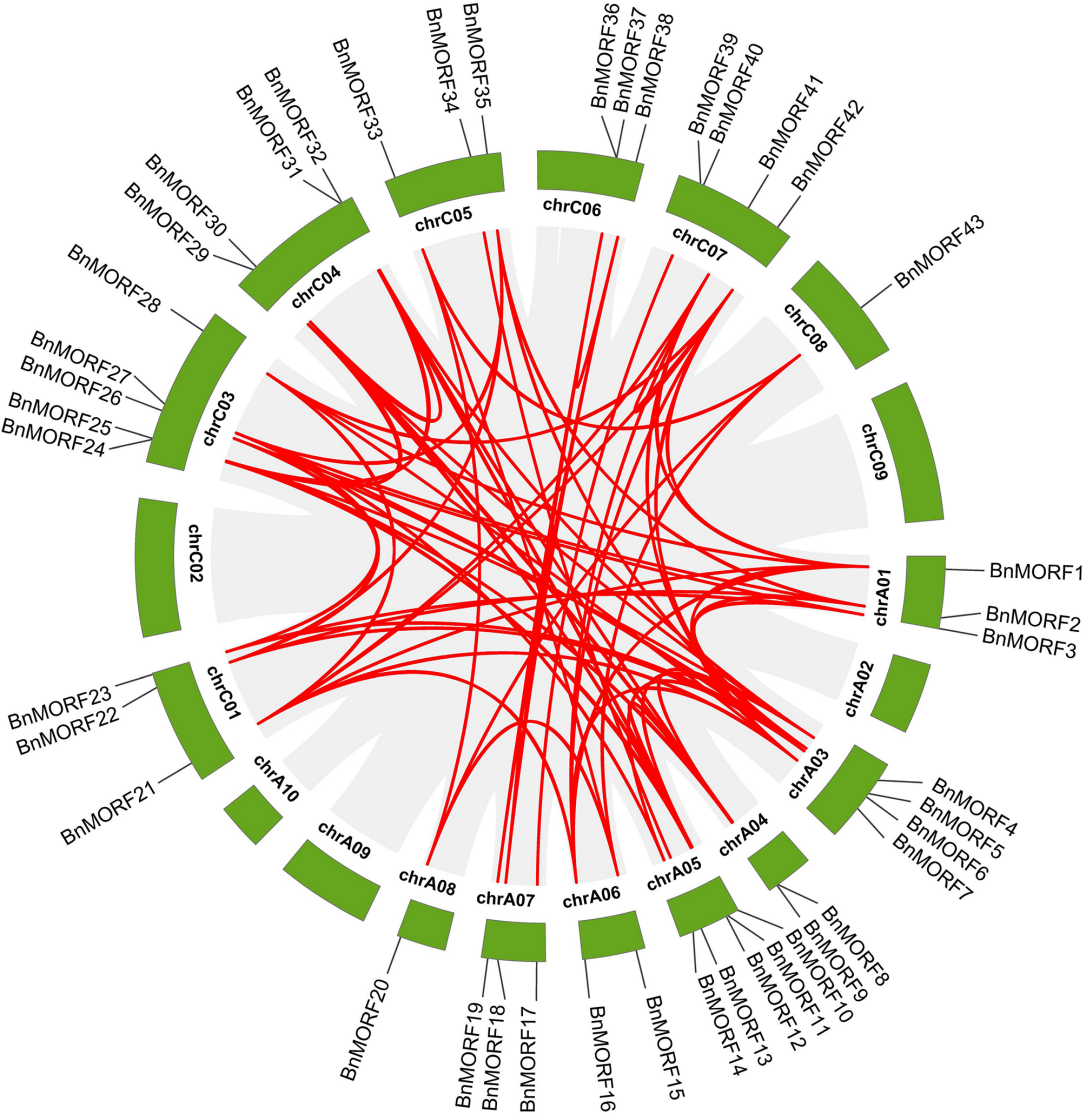
Intra-species collinearity analysis of BnMORF family members. *BnMORF* genes are mapped to 14 *Brassica napus* chromosomes in a circle, and segmental duplications are mapped to their respective locations. The red lines represent the homologous relationships among *BnMORF* family members, and the gray lines represent all background collinear pairs

The chromosomal localization analysis revealed that 43 *BnMORF* genes were scattered irregularly across 14 chromosomes. The number of genes on each chromosome has no bearing on chromosome size (Fig. 3). No *BnMORF* genes are located on chromosomes A02, A09, A10, C02, and C09. There are 20 *BnMORF* genes located on the A subgenome, while the rest of the *BnMORF* genes are located on the C subgenome. Chromosomes A05 and C03 both contain the largest number of five *BnMORF* genes, while chromosomes A08 and C08 both contain only one *BnMORF* gene (Fig. 3). To investigate the evolution of *BnMORF* genes, the synteny between *Brassica napus* and *Arabidopsis* at the whole genome level was analyzed. Between the two genomes, 40 collinear *MORF* gene pairs were identified (Fig. 4, Additional file 2: Table S1). All *Arabidopsis MORF* (*AtMORF*) genes have multiple syntenic *BnMORF* genes. For example, both *AtMORF1*, *AtMORF4*, and *AtMORF8* have six collinear *BnMORF*s. However, *AtMORF5* has only two synthetic *BnMORF*s (Additional file 1: Table S1). We also investigated the duplication events of *BnMORF* family genes by BLASTP and MCScanX. We discovered that *BnMORF* genes were mainly derived from whole genome duplication and segmental duplication events (Additional file 3: Table S2). In addition, intra-species collinearity analysis of 43 *BnMORF* genes revealed 76 collinear gene pairs (Fig. 5, Additional file 4: Table S3). These results indicated that segmental duplication and whole genome duplication appeared to play a major role in the expansion of the *BnMORF* gene family.

### The *cis*-regulatory elements and transcription factor binding sites analysis in promoters of *BnMORF* genes

**Fig. 6:**
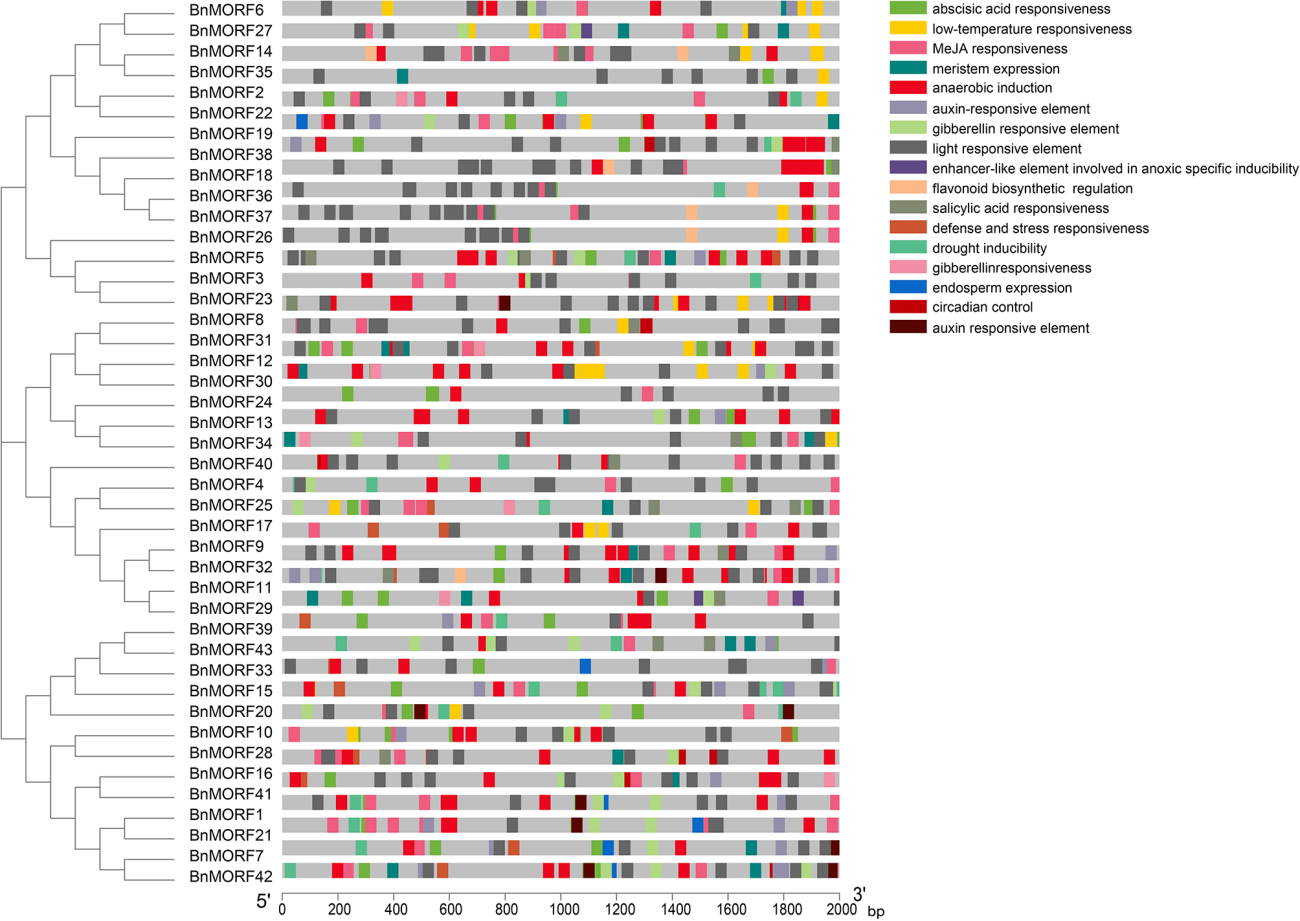
Predicted *cis*-regulatory elements in promoters of *BnMORF* genes. On the right side, the names of each *cis*-regulatory element are displayed. Different colored boxes in the gray rectangle represent different *cis*- regulatory element. The scale can be utilized for estimating each element’s relative position. The direction of promoter sequence is from 5’ to 3’

**Fig. 7:**
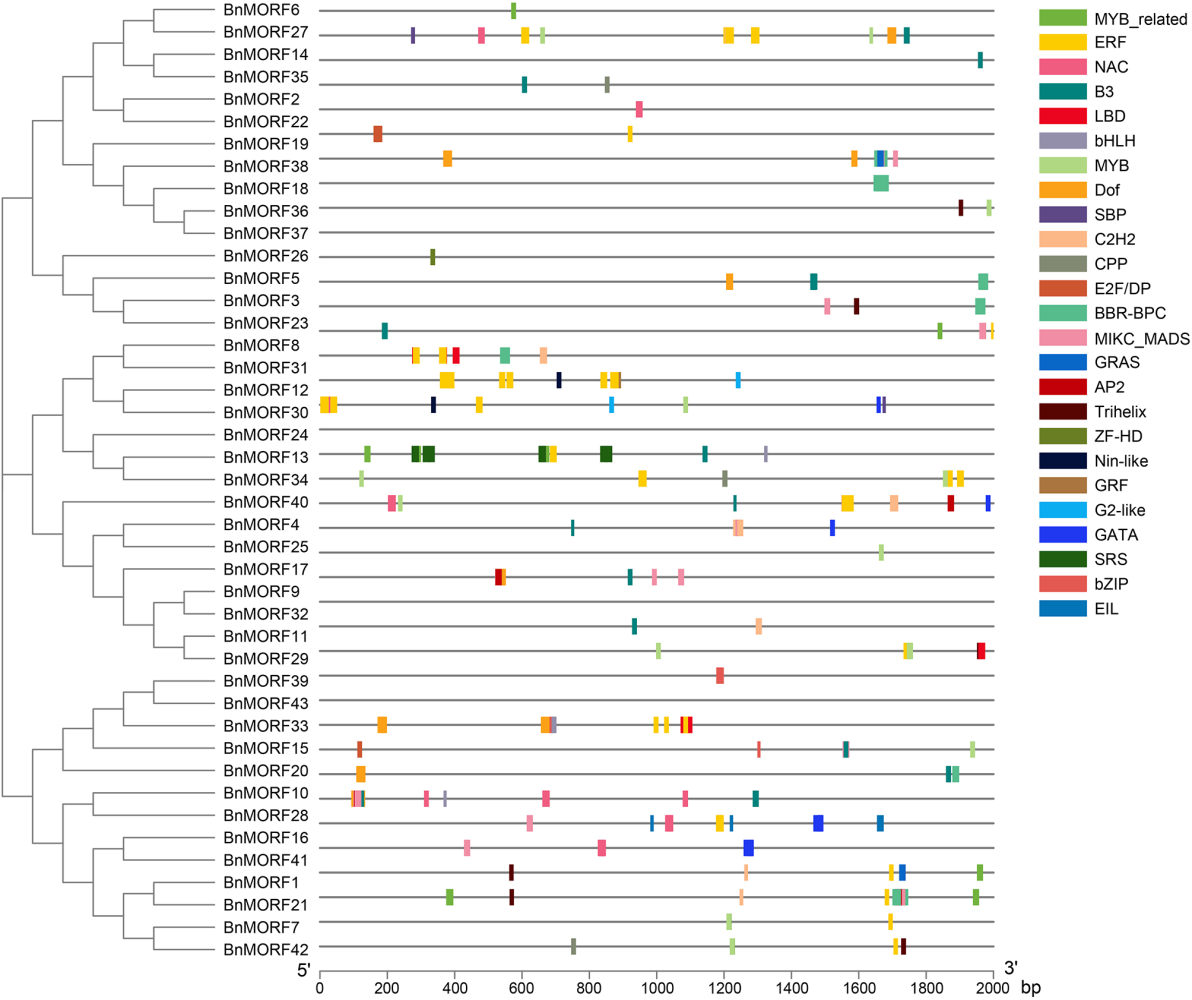
Predicted transcription factor binding sites in *BnMORF* promoters. Boxes of different colors represent the binding sites of different transcription factors. The names of various transcription factor families are shown on the right

*Cis*-regulatory elements and transcription factor binding sites are crucial in modulating gene expression, and promoters of genes with related functions may contain similar regulatory elements. We also carried out the *cis*-regulatory elements and transcription factor binding sites analysis in promoters of *BnMORF* genes. As displayed in Fig. 6, the identified *cis*-regulatory elements in the promoters of *BnMORF* genes can be classified into four main categories. The first category is for light reactions. The promoters of all *BnMORF* genes contained several light-responsive elements, the majority of which were AE-box elements. The elements of development and growth, including circadian regulation, endosperm expression, and meristem expression, were covered in category two. Abscisic acid, auxin, gibberellin, methyl jasmonate, and salicylic acid were the members of category three. At least one *cis*-regulatory element involved in phytohormone responsiveness classification was found in all *BnMORF* genes. Further investigation revealed that 37 genes carried methyl jasmonate responsive elements, 35 carried abscisic acid responsive elements, 21 carried auxin responsive elements, 14 carried salicylic acid responsive elements, and 27 genes carried gibberellin responsive elements (Additional file 5: Table S4). Category four is associated with abiotic stresses such as drought inducibility, low-temperature, and anaerobic induction. There were *cis*-regulatory elements for anaerobic induction in 38 genes, for low-temperature responsiveness in 21 genes, and for drought induction in 20 genes, indicating that *BnMORF* genes may be factors responding to abiotic stresses. Binding sites of 25 TF families, including Trihelix, ERF, GRAS, C2H2, MYB_related, BBR-BPC, B3, Dof, Nin-like, LBD, GRF, G2-like, bHLH, bZIP, GATA, NAC, EIL, MIKC_MADS, MYB, E2F/DP, AP2, CPP, SBP, SRS, ZF-HD, and HD-ZIP, were identified in promoters of *BnMORF* genes (Fig. 7). The ERF family covers 16 BnMORF members, while the SRS and ZF-HD families cover only one family member, respectively (Fig. 7).

### The expression pattern of *BnMORF* genes at different developmental stages

**Fig. 8:**
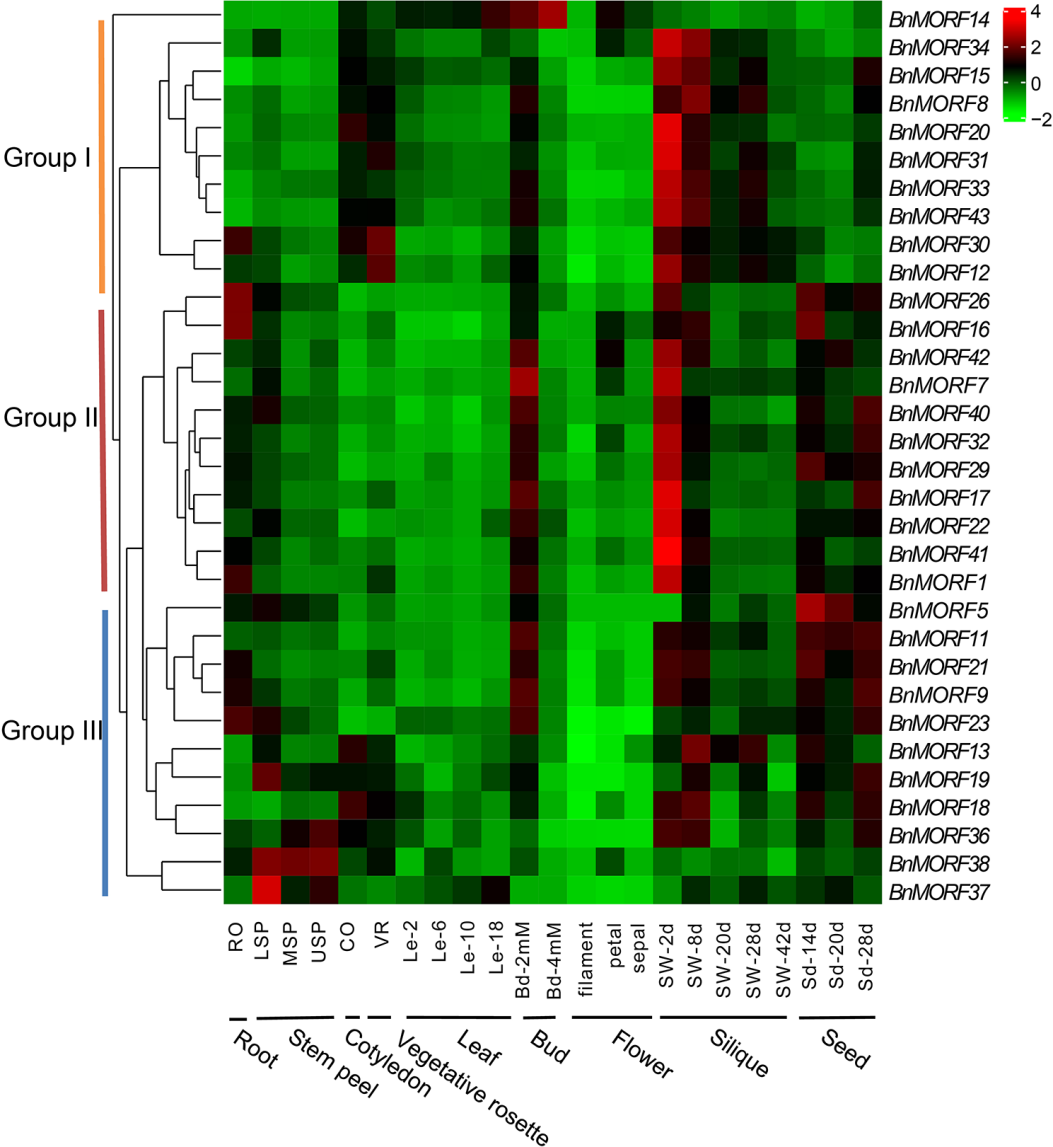
The expression pattern of *BnMORF* genes in different tissues or organs of *Brassica napus* cultivar ZS11 at different developmental stages. The expression profile of the 32 *BnMORF* genes are displayed in a hierarchical cluster. At the bottom of each column, tissues or organs from various developmental phases of *Brassica napus* cultivar ZS11 employed for expression profiling are noted. On the left is a cluster dendrogram. The color key represents the Z-score values transformed from the expression values. Ro: root; LSP: lower stem peel; MSP: middle stem peel; USP: upper stem peel; Le: leaf; Co: cotyledon; VR: vegetative rosette; Bd: bud; Si: silique; SW: silique wall; Sd: seed; d indicates day

The study of a gene’s expression pattern in various tissues or organs will help to unravel its biological function. The BnIR database was utilized for obtaining the expression data of *BnMORF* genes in different tissues or organs (root, stem, cotyledon, vegetative rosette, leaf, bud, flower, silique, and seed) throughout various developmental stages. Excluding for 11 genes that showed no detectable expression levels (TPM < 0.5) in any of the samples tested, the remaining 32 genes had obvious preferential expression patterns (Additional file 6: Table S5). The 32 *BnMORF* genes are clustered into three groups based on their expression patterns (Fig. 8). Nearly all the *BnMORF* genes have relative high expression in the bud and silique of early development stages. Furthermore, in group I, all 10 genes preferentially express in the cotyledon, vegetative rosette, and young silique, except that *BnMORF14* shows obvious leaf and bud-specific expression. In group II, most members also show relative high expression in root and seed, and *BnMORF16* and *BnMORF26* display specific high expression in root and young seed. In group III, most genes have relative high expression in seed, except that *BnMORF37* and *BnMORF38* present stem-specific expression (Fig. 8).

### The expression profile of *BnMORF* genes in response to different stress and phytohormone treatments

**Fig. 9:**
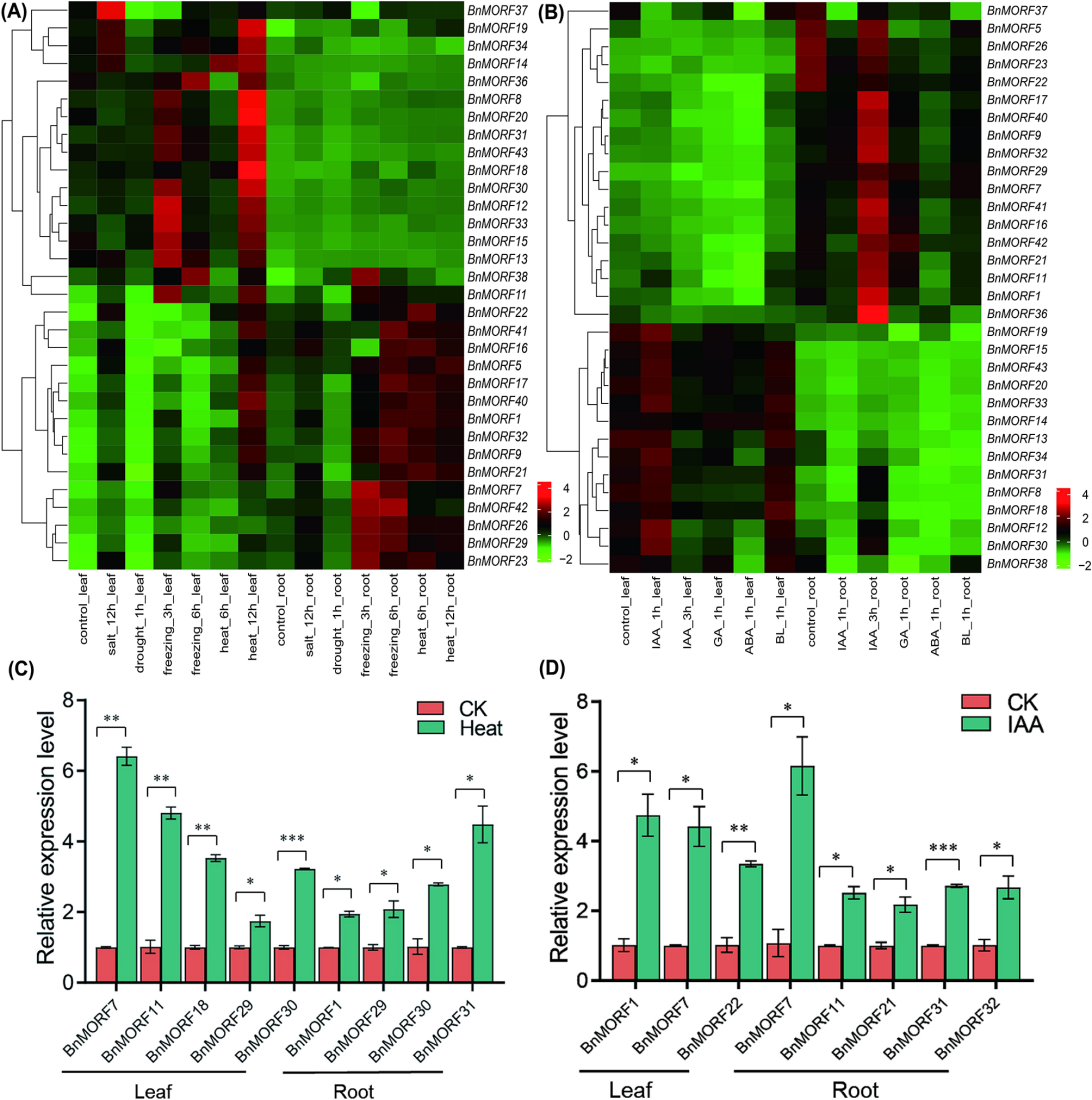
The expression profile of *BnMORF* genes under different stress and phytohormone treatments. **(A)** The expression pattern of *BnMORF* genes in leaf and root under salt, drought, freezing, and heat treatments at different time points. **(B)** The expression pattern of *BnMORF* genes in leaf and root treated phytohormone with IAA, GA, ABA, and BL at different time points. Z-score normalization was used to process the expression data. The color scale represents relative expression levels from low (green) to high (red). h indicates hour. **(C)** The expression level of *BnMORF* genes revealed by RT-qPCR in leaves and roots under heat treatment. Data are mean ± SEM from two biological replicates, and asterisks denote significant differences using a two-tailed Student’s *t* test (*p-value < 0.05, **p-value < 0.01, ***p-value < 0.001). **(D)** The expression level of *BnMORF* genes revealed by RT-qPCR in leaves and roots under IAA treatment. Data are mean ± SEM from two biological replicates, and asterisks denote significant differences using a two-tailed Student’s *t* test (*p-value < 0.05, **p-value < 0.01, ***p-value < 0.001)

We also investigated the modulation of *BnMORF* gene expression in response to various abiotic stress and phytohormone treatments (Additional file 7: Table S6). The expression of *BnMORF* genes of groups I and III (except *BnMORF36* and *BnMORF37*) classified by different developmental stages (Fig. 8) was preferential induced in leaves other than in roots, while the accumulation of transcripts of genes of group II was more abundant in roots (Fig. 9a). We discovered that the transcript levels in leaves of most *BnMORF* genes except *BnMORF37* were significantly induced (more than 2-fold) after being exposed to heat for 12 h, and the expression of *BnMORF* genes belonging to group II (except *BnMORF42*) in roots was more responsive to heat stress in roots. In addition, the expression of *BnMORF* genes in group I (except *BnMORF14* and *BnMORF34*) was significantly increased in the leaves with 3 h of freezing treatment. The expression in the roots of *BnMORF32*, *BnMORF7*, *BnMORF26*, and *BnMORF29*, which belonged to group II, was also strongly induced. Notably, *BnMORF37*, *BnMORF19*, *BnMORF34*, and *BnMORF14* had low expression levels in leaves under normal conditions, but were considerably up-regulated in leaves under salt stress, indicating potential involvement in leaves in response to salt stress (Fig. 9a). These expression patterns suggested that BnMORF members may have significant response functions to abiotic stress, especially heat and cold stress. We also found that gibberellin (GA) and abscisic acid (ABA) treatment all decreased the expression of group I genes in leaves and the expression levels of group II genes in roots (Fig. 9b). Surprisingly, the mRNA accumulation in group I *BnMORF* genes (except for *BnMORF14*) in leaves fluctuated obviously, exhibiting increased expression after IAA treatment. The expression of group II *BnMORF* genes (except *BnMORF22* and *BnMORF26*) in roots increased significantly after three hours of IAA treatment. We subsequently selected 10 *BnMORF* genes exhibiting significant expression level changes in response to heat and IAA treatment and used quantitative reverse transcription PCR (RT-qPCR) to validate their expression patterns. The results showed that the expression levels of these 10 *BnMORF* genes were significantly up-regulated in leaves or roots after heat or IAA treatments (Fig. 9c and d), which was consistent with the above expression profile obtained from the BnIR transcriptome database.

**Fig. 10:**
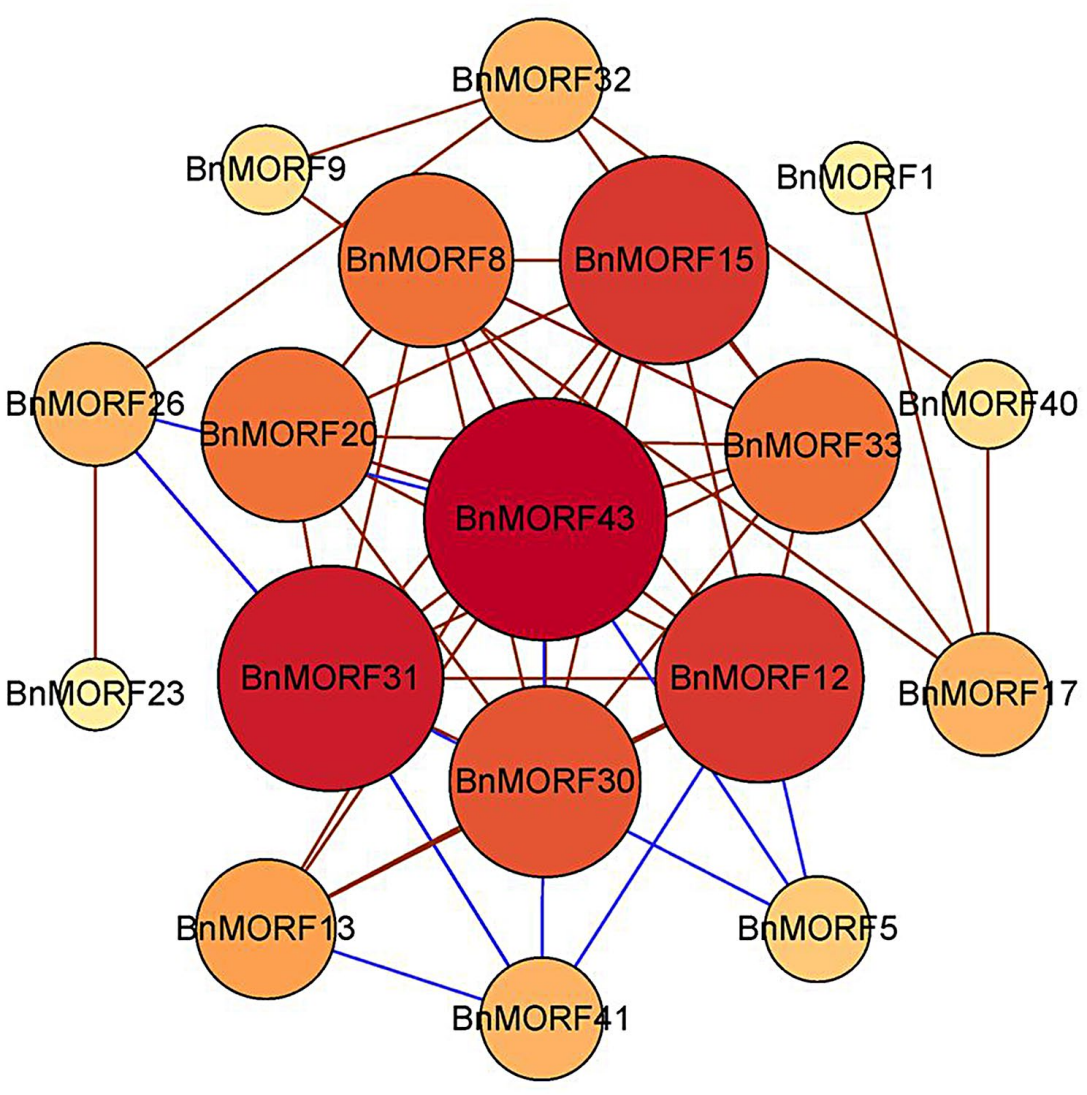
The co-expression network of *BnMORF* genes in response to different stresses. Each gene’s hue is determined by its degree and betweenness centrality. The red genes indicate strongly connected nodes, whereas the yellow genes indicate less connected nodes. Positive and negative correlations are represented by the red and blue lines between genes, respectively

A co-expression network of *BnMORF* genes responding to different stresses was also constructed (Fig. 10). *BnMORF43* was the top hub gene in the network with the highest degree and betweenness centrality, with 11 *BnMORF* genes directly linked to it. Interestingly, the co-expression genes such as *BnMORF43*, *BnMORF30*, *BnMORF12*, *BnMORF33*, and *BnMORF20* had the same expression pattern across the tissues and in response to different abiotic stress and hormone treatments (Figs. 8 and 9). These results indicate that these *BnMORF* genes may be co-regulated, functionally linked, or involved in the same signaling pathway or physiological activity.

## Discussion

In general, RNA editing in plants is considered a DNA mutation correction process at the RNA level, restoring conserved amino acids to guarantee that proteins continue to function normally [15]. Plant MORF proteins are multifunctional proteins that interact with other RNA editing factors and play an essential role in chloroplast gene editing [7]. Moreover, recent studies demonstrated that *MORF* genes were important for growth of plants and responses to stressful conditions, including survival of seeds in rice [16], pathogen stress in tobacco and kiwifruit [18, 26], and drought-related stress in poplar [5]. However, no research on the *MORF* gene family in *Brassica napus* has been documented. We identified 43 BnMORF family members in *Brassica napus* using the recently published ZS11 genome. Subcellular localization prediction revealed that 63% of BnMORF members were localized to mitochondria and 23% to chloroplasts, which was consistent with the fact that most MORF proteins in *Arabidopsis* are mitochondrion localization and a few are localized to chloroplasts [10]. Subcellular localization prediction revealed that four homologs of AtMORF2 in *Brassica napus*, BnMORF8, BnMORF12, BnMORF30, and BnMORF31, are localized to chloroplasts. Moreover, the corresponding genes of these four BnMORF proteins were highly expressed in green tissues such as the vegetative rosette and siliques. The gene structure suggested that most BnMORF members within the same phylogenetic tree clan presented similar exon-intron distribution patterns as well as motif compositions. We hypothesized that the differences in physical and chemical properties of BnMORF proteins might be caused by the diversity of gene structure and motif compositions.

Gene replication plays an essential role in the evolution of species, mainly through whole genome replication, segmental and tandem replications [27]. *Brassica napus* developed naturally from the hybridization of two diploid species, and its diploid parents also experienced *Arabidopsis*-based polyploidization events [24]. In theory, after genome-wide polyploidy, *Brassica napus* should have six homologous genes for each *Arabidopsis* gene. However, only 43 BnMORF family members were identified in this study, suggesting that about 20% of *MORF* genes had been lost during evolution. Although *AtMORF* genes have multiple direct homologs in *Brassica napus*, only *AtMORF1*, *AtMORF4*, and *AtMORF8* had six homologous genes, according to the interspecific collinear study of *Arabidopsis* and *Brassica napus*. The results showed that other *AtMORF* homologous genes experienced complicated events such as gene expansion and loss during the evolution of *Brassica napus*. The collinearity analysis also showed that most BnMORF family members were generated by whole genome duplication or segmental duplication. The *MORF* genes in the *Brassica napus* genome were expanded to some extent even if gene loss happened during the evolution of *Brassica napus*, indicating that gene duplication played a significant role in the development of *Brassica napus*.

It was hypothesized that tissue-specific expression of *MORF* genes would determine their functional divergence. Whole growth period expression profiling analysis of *BnMORF* genes revealed that most *BnMORF* genes were universally expressed in green tissues such as buds and young siliques. Some genes were expressed in stems and roots specifically, indicating the expanded function of *BnMORF* genes. These results indicated that *BnMORF* genes may function closely in various tissues and organs. In addition, we found that *BnMORF* genes that are closely related in evolution have similar expression patterns. An increasing number of studies have shown that RNA editing events in chloroplast and mitochondrial genes play an important role in response to a variety of environmental stresses. Low temperatures affect the processing of wheat mitochondrial *cox2* transcripts and reduce the efficiency of RNA editing [28]. When maize was exposed to 37℃, the editing efficiency of *rps14* and *rpl20* transcripts decreased by 70% [29]. In different strains of rice, more than half of the 90 editing sites in transcripts of six mitochondrial genes were sensitive to oxidative stress treatment, and the editing efficiency of these sites were modified by oxidative stress [30]. We discovered that promoters of *BnMORF* genes possessed a variety of *cis*-regulatory elements and transcription factor binding sites associated with abiotic stress and phytohormone responses. Moreover, the expression of many *BnMORF* genes was regulated by different abiotic stress and hormone treatments. This implied that *BnMORF* genes might also been engaged in the development and growth of rapeseed by responding distinct abiotic stress and phytohormone regulatory pathways through their roles in chloroplast and mitochondrial RNA editing.

## Conclusions

In this study, 43 *BnMORF* genes were identified from the latest annotated version of the *Brassica napus* cultivar “Zhongshuang 11” genome. Phylogenetic analysis divided *BnMORF* gene family members into three groups, and this classification was further supported by similar conserved motif compositions and exon-intron distributions. The collinearity analysis showed that segmental and whole genome duplication had a significant impact on the expansion of the *BnMORF* gene family. A number of *cis*-regulatory elements and transcription factor binding sites related to abiotic stress and hormone responses were also identified in promoters of *BnMORF* genes. Transcriptome data also revealed that *BnMORF* genes might participate in growth and development, as well as abiotic stress and hormone responses. Taken together, these findings provide a foundation for further exploring the functions of *MORF* genes in *Brassica napus*.

## Methods

### Identification of MORF family members in *Brassica napus*

Taking the *Brassica napus* cultivar “Zhongshuang 11” (ZS11) ZS11.v10 genome as a reference, the candidate BnMORF family members were identified by BLASTP search in the *Brassica napus* genome database (http://yanglab.hzau.edu.cn/BnIR/BLAST/) [31] using MORF protein sequences from *Arabidopsis* as queries and the e-value ≤ 5 as a threshold. We retrieved the protein sequences and gene model annotation files from BRAD (http://www.brassicadb.cn/). InterProScan (http://www.ebi.ac.uk/interpro/) was further used to confirm the existence of the MORF domain (IPR039206) in candidate proteins. Sequences without MORF domains were removed. According to the chromosomal locations of corresponding genes, the remaining BnMORF members were named sequentially. The molecular weight and isoelectric point of BnMORF proteins were calculated using the protparam (https://web.expasy.org/protparam/) [32] In addition, subcellular localization and chloroplast/mitochondrion transit peptides of BnMORF proteins were predicted by TargetP2.0 (https://services.healthtech.dtu.dk/services/TargetP-2.0/) and PredSL (http://bioinformatics.biol.uoa.gr/).

### Phylogenetic tree construction

In addition to *Brassica napus*, we also used MORF members from three more species (*Arabidopsis thaliana*, *Oryza sativa*, and *Zea mays*) for phylogenetic analysis to explore the function and molecular evolution of BnMORF members. The muscle method was utilized to align the MORF protein sequences, and the tree of phylogenetic relationships was built in MEGA7 software using the neighbor-joining method with the Possion model, 1000 bootstrap values, and pairwise deletion [33]. Besides, the phylogenetic tree among MORF members in *Brassica napus* was also constructed using the above method. The phylogenetic trees were further embellished using ITOL (http://itol.embl.de/).

### Conserved motif and gene structure analysis

The predicted introns and exons in *BnMORF* genes were extracted from the GFF3 file of *Brassica napus* ZS11.v10, and their intron and exon structures were visualized by Gene Structure View of TBtools software. We explored the conserved motifs in BnMORF proteins utilizing the MEME motif suite (https://memesuite.org/meme/index.html/) [34] and used the TBtools to visualize them [35].

### Chromosomal location and collinearity survey of *BnMORF* genes

*BnMORF* genes were mapped to the *Brassica napus* genome using MG2C (http://mg2c.iask.in/mg2c_v2.1/*).* Chromosome length, gene location, and length information from the GFF3 annotation file were extracted by TBtools. The synteny links between orthologous *Arabidopsis* and *Brassica napus MORF* genes were determined using the dual-synteny-plotter function of TBtools. With default options, the Multiple Collinearity Scan Toolkit (MCScanX) was used to evaluate the gene duplications of the BnMORF family members, and Circos (http://circos.ca/) was used to display the collinearity information [36, 37].

### Spatial-temporal and abiotic stress expression profile analysis of *BnMORF* genes

The expression data of *BnMORF* genes was obtained from the expression profile (ZS11 library) of BnIR. The genes with TPM (transcripts per million) < 0.5 were excluded from further analysis. Z-score normalization was used to process the expression data and heatmaps were generated by TBtools. The co-expression analysis was performed using the Pearson correlation coefficient and visualized by Cytoscape [38, 39]. We obtained 14 RNA-seq datasets on *BnMORF* gene responses to different stresses from the BnIR expression profile (ZS11 library) for co-expression analysis.

### Promoter analysis and transcription factor binding site prediction

Genomic DNA sequences up to 2000 bp upstream of the start codon of each *BnMORF* gene were extracted from the *Brassica napus* multi-omics information resource database (BnIR, https://yanglab.hzau.edu.cn/), and then PlantCare software (http://bioinformatics.psb.ugent.be/webtools/plantcare/html/) was used to predict *cis*-regulatory elements in the promoter region of *BnMORF* genes. In addition, we use the PlantTFDB website (http://planttfdb.cbi.pku.edu.cn/) to predict transcription factor binding sites with a setting of *p*-value ≤ 1e^− 6^. TBtools software was used to visualize the number and distribution of *cis*-regulatory elements and transcription factor binding sites.

### RT-qPCR analysis of *BnMORF* genes under heat and IAA treatments

The expression level of *BnMORF* genes under heat and IAA treatments was examined by RT-qPCR. *Brassica napus* ZS11 plants were grown on 1/2 LS solid medium under long-day conditions with 100 µmol·m^− 2^·s^− 1^ light intensity at 22 °C for 14 days. For heat treatment, seedlings were placed at 38 °C for 3 h, followed by recovery at 22 °C for 12 h. For IAA treatment, 14-day-old seedlings were treated with 10 µM IAA for 3 h. Following the treatment, root and leaf samples were collected, respectively. Root and leaf samples collected from untreated plants were served as controls [31]. Total RNA was isolated using the RNAprep Pure Plant Kit (DP432, Tiangen, China). The first-strand cDNA was synthesized using the HiScript III 1st Strand cDNA Synthesis Kit (+ gDNA wiper) (R312-02, Vazyme, China) with the addition of Oligo (dT)_20_VN and random hexamer primers. The primers used in RT-qPCR analysis were listed in Additional file 8: Table S7. RT-qPCR was performed using the Bio-rad CFX Connect Real-Time PCR Detection System and the ChamQ Blue Universal SYBR qPCR Master Mix (Q312-02, Vazyme, China). *PP2A* (Protein phosphatase 2 A subunit A3), which showed suitability across multiple conditions, was used as the reference gene [40]. The relative expression level of each gene was calculated by the 2^−ΔΔCt^ method [41].

### Electronic supplementary material

Below is the link to the electronic supplementary material.

Supplementary Material 1

Supplementary Material 2

Supplementary Material 3

Supplementary Material 4

Supplementary Material 5

Supplementary Material 6

Supplementary Material 7

Supplementary Material 8

Supplementary Material 9

## Data Availability

All the data supporting the results of this article is included in the article and additional files.

## Abbreviations

MORF: Multiple organellar RNA editing factor
BnMORF: *Brassica napus* MORF
RIP: RNA editing factor interacting protein
ZS11: Zhongshuang 11
pI: Isoelectric point
aa: Amino acids
TF: Transcription factor
TPM: Transcripts per million
RNA-seq: RNA sequencing
