# Supplementary material for: Comprehensive identification, characterization, and expression analysis of the MORF gene family in Brassica napus
Source: BMC Plant Biol. 2024 May 30;24:475. doi: 10.1186/s12870-024-05177-3 (PMC11138011; doi:10.1186/s12870-024-05177-3)
Supplement: Supplementary file 1 — Supplementary Material 1 [file 12870_2024_5177_MOESM1_ESM.pdf]

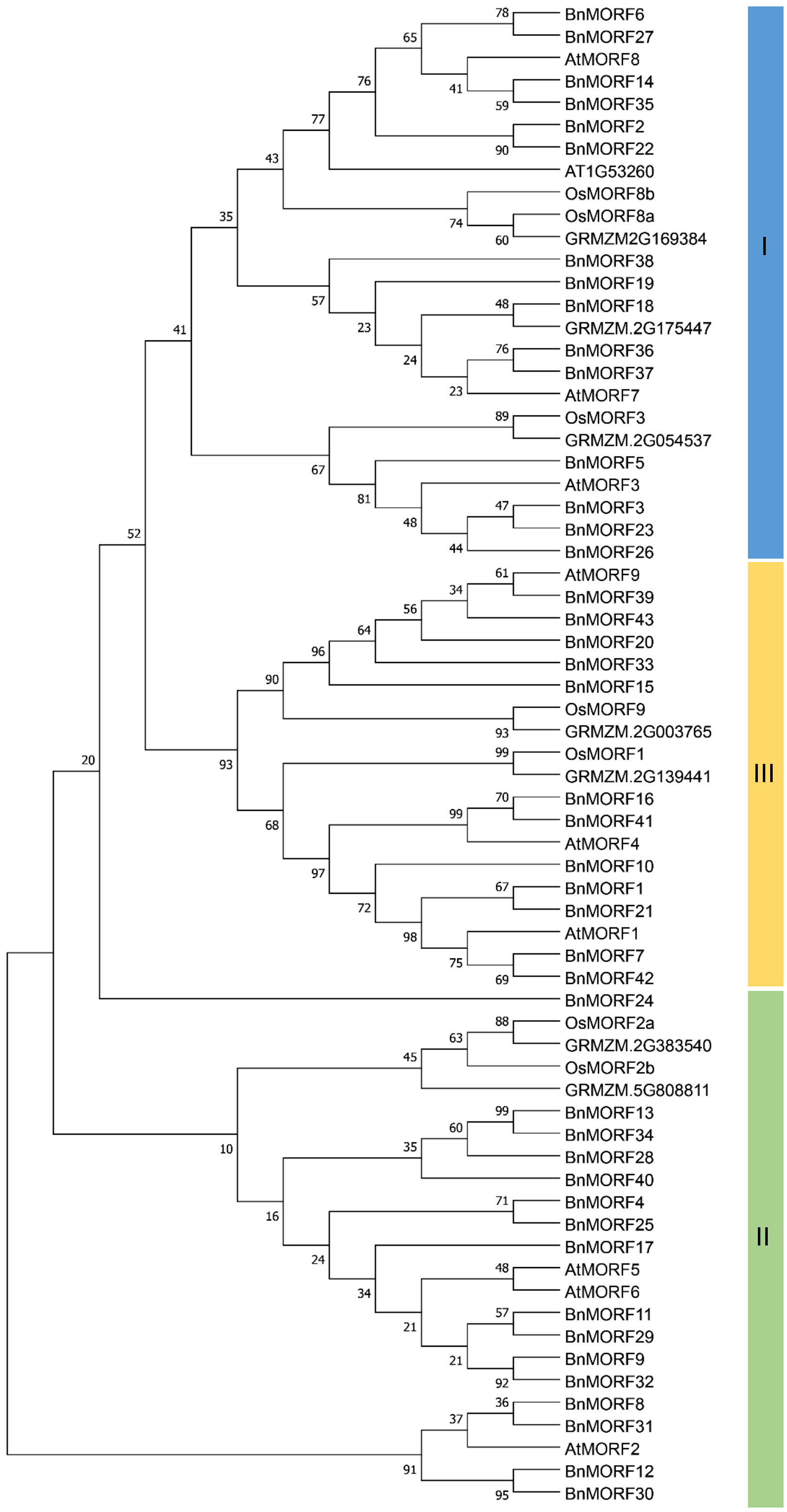

Fig. S1 Phylogenetic relationships of the *MORF* family members from *Brassica napus*, *Oryza sativa*, *Arabidopsis thaliana*, and *Zea mays*. The full-length amino acid sequences were used for phylogenetic tree construction by the neighbor-joining (NJ) method with MEGA 7.0 software. All the MORF members were classified into three groups and designated as Group I - III. Branches from different groups are indicated by different colors, the Bootstrap values are indicated on the branches.
