# Supplementary material for: Comprehensive identification, characterization, and expression analysis of the MORF gene family in Brassica napus
Source: BMC Plant Biol. 2024 May 30;24:475. doi: 10.1186/s12870-024-05177-3 (PMC11138011; doi:10.1186/s12870-024-05177-3)
Supplement: Supplementary file 9 — Supplementary Material 9 [file 12870_2024_5177_MOESM9_ESM.docx]

**Supplementary Information**

**Additional file 1: Fig. S1**. Phylogenetic tree of the *MORF* family members from *Brassica napus*, *Oryza sativa*, *Arabidopsis thaliana*, and *Zea mays*.

**Additional file 2: Table S1**. Interspecies collinear gene pairs.

**Additional file 3: Table S2**. Duplication type of *BnMORF* family members in *Brassica napus*.

**Additional file 4: Table S3**. List of syntenic blocks of the *BnMORF* family members in *Brassica napus*.

**Additional file 5: Table S4**. The *cis*-regulatory elements found in the promoter regions of *BnMORF* genes.

**Additional file 6: Table S5**. The expression profile of *BnMORF* genes across different tissues.

**Additional file 7: Table S6**. The expression profile of *BnMORF* genes in response to different stress and phytohormone treatments.

**Additional file 8: Table S7**. Primers used in RT-qPCR to verify the expression levels of *BnMORF* genes under heat and IAA treatments.
